# Pelvic ultrasound finding of late-onset bladder erosion after transobturator tape for female stress urinary incontinence: A case report

**DOI:** 10.1097/MD.0000000000033129

**Published:** 2023-03-03

**Authors:** Yelin Lou, Yang Hu, Yibo Zhou

**Affiliations:** a Department of Ultrasonography, Affiliated Jinhua Hospital, Zhejiang University School of Medicine, Jinhua, China; b Department of Urology, Affiliated Jinhua Hospital, Zhejiang University School of Medicine, Jinhua, China.

**Keywords:** continence surgery, ultrasound, urinary incontinence

## Abstract

Mid-urethral sling is a gold-standard procedure for stress urinary incontinence because of its high efficacy and low complication incidence. Moreover, mesh erosion into the bladder is a rare complication.

**Patient concerns:**

The 63-year-old patient visited our gynecology clinic with complaints of gross hematuria and was diagnosed with bladder erosion by ultrasound 6 months after transobturator tape procedure.

**Diagnoses:**

The 2D ultrasound found the sling in the bladder wall perforation, which can lead to the formation of bladder stones. Meanwhile, 3D ultrasound showed the left side of the sling crossed the bladder mucosa at 5 o’clock.

**Interventions:**

The sling and bladder stones were removed by holmium laser.

**Outcomes:**

The patient underwent a follow-up pelvic ultrasound at 6 months, which showed no erosion mesh under the bladder mucosa.

**Lessons:**

Pelvic ultrasound could accurately evaluate the location and shape of the tape, which is important for a reasonable surgical plan.

## 1. Introduction

Stress urinary incontinence (SUI) is characterized by uncontrollable urine leakage, which is associated with a sudden increase in abdominal pressure due to coughing, sneezing, effort, or exertion. SUI seriously affects the physical and mental health and quality of life of patients. The prevalence rate of SUI in adult women is 15.7%, and 77.5% of patients suffer from urinary incontinence symptoms, of which 28.8% suffer from moderate to severe distress related to the severity of SUI.^[1]^ Mid-urethral vaginal suspension surgeries, including retropubic tension-free vaginal tape (TVT) and transobturator tape (TOT), are successful surgical methods for SUI, with over 90% patient satisfaction rates.^[2]^ Compared with TVT, TOT is characterized by simple operation, less trauma and no routine cystoscopy during the procedure. However, there are increasing reports of complications after TOT such as difficulty in urination, recurrence of urinary incontinence, pain and discomfort in the inner thigh, obturator hematoma, and sling erosion of the vagina, urethra, or bladder. Bladder erosion by sling is a rare complication, and there are no specific cases reported to date.^[3]^ Herein, we reported the diagnosis of bladder erosion in a patient using pelvic floor ultrasound (PFU) examination and an optimal strategy for management of bladder erosion after TOT.

## 2. Case presentation

A 63-year-old Chinese woman presented to our gynecology clinic with complaints of postoperative pain in the lower abdomen, gross hematuria, frequency, and urgency. She had a history of laparoscopic total bilateral adjunctive hysterectomy and TOT 6 months ago.

The patient was a homemaker, with no history of drinking or smoking. She had 3 deliveries and all of them were vaginal births. She had hypertension for 8 years and the routine use of brimonidine 5 mg/Qd resulted in moderate blood pressure control. She had a history of cerebral infarction and had been taking aspirin regularly. There was no similar medical history in her family.

**Figure 1.**
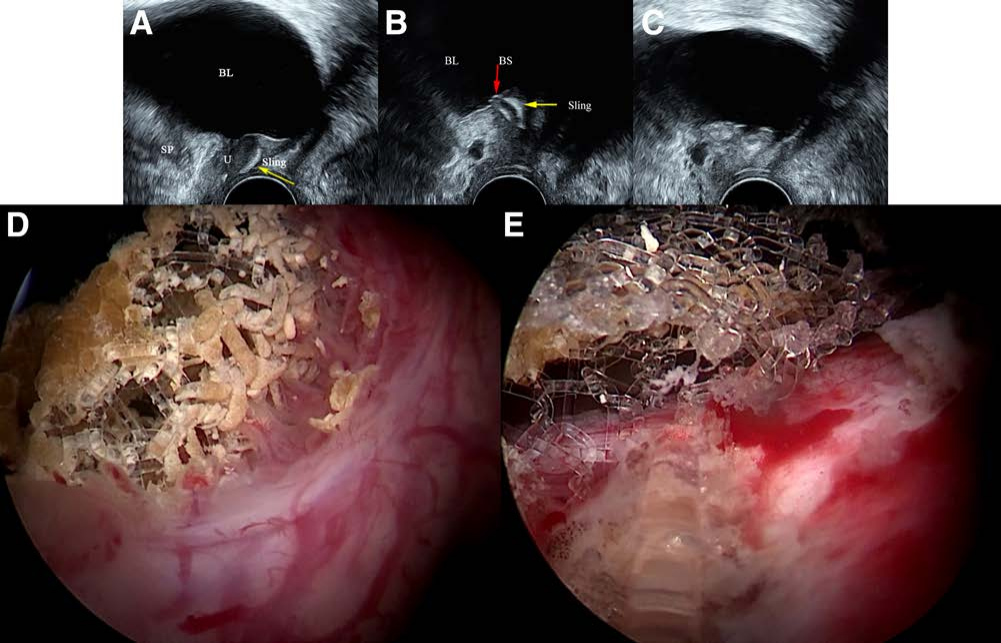
(A–C) Two-dimensional (2D) PFU images were obtained with the translabial method using a 3.5-MHz sector probe. Sagittal views were obtained at rest. The bladder (BL), bladder stones (BS), urethra (U), and sling were seen. The yellow arrows indicate the sling and the red arrow shows the bladder stones. (D and E) Images show mesh erosion into the urinary bladder and the formation of secondary bladder stones, which were resected using a holmium laser by cystoscopy. PFU = pelvic floor ultrasound.

**Figure 2.**
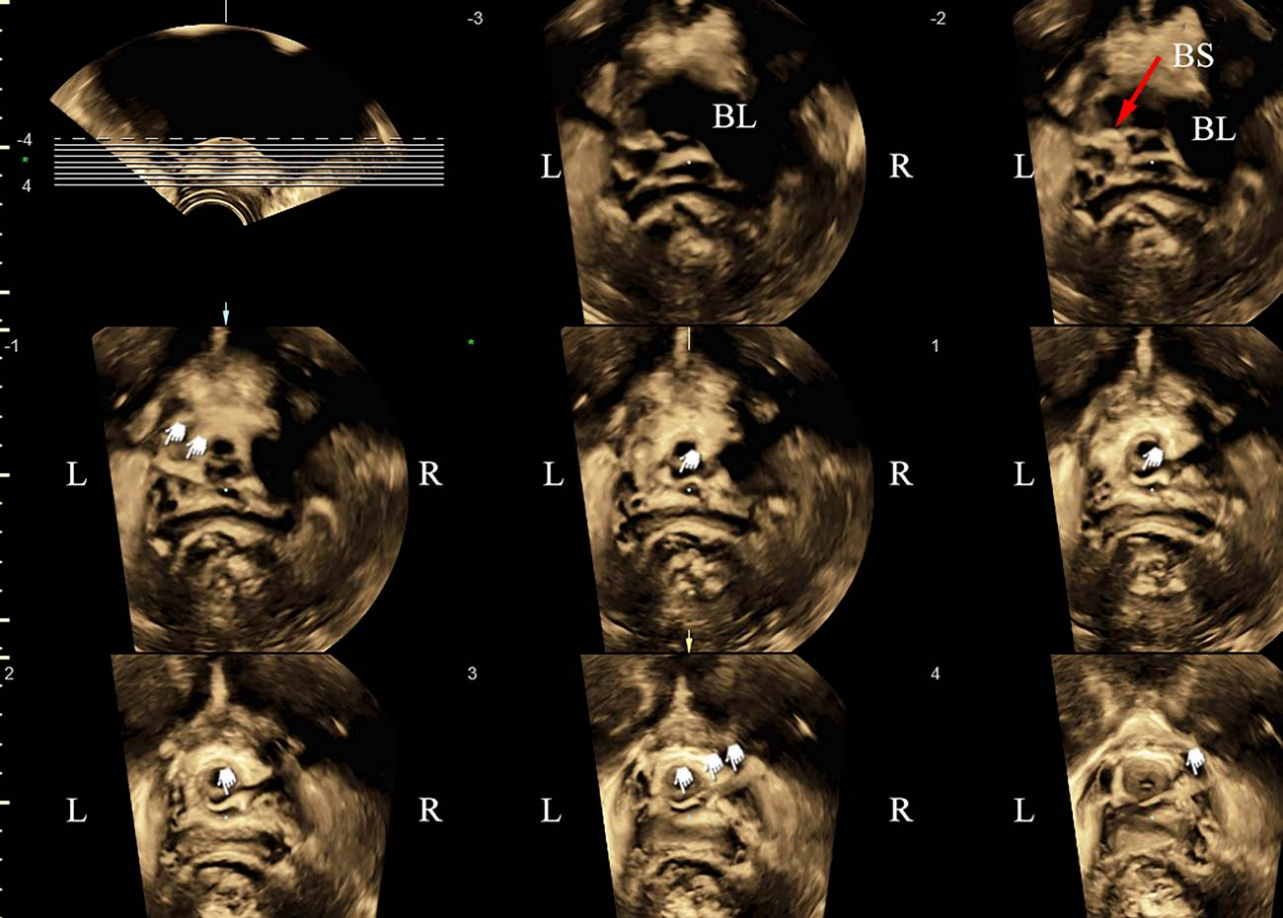
Three-dimensional (3D) ultrasound shows 8 tomographic images. The red arrow indicates the bladder stones. The white hand shapes show the track of sling. BL = bladder, BS = bladder stones, L = left, R = right.

On admission, her temperature was 36.7 °C, pulse was 71 beats/min, and blood pressure was 127/80 mm Hg. She was conscious and alert. She was psychologically depressed. In the routine physical examination, no abdominal tenderness or anatomical abnormalities were detected. Auscultation of her heart indicated no murmurs or arrhythmia. Respiratory frequency was 18/min, and no wheezing or rales were found. Neurological examination indicated that her muscle strength and tension were normal. A urogynecological physical examination showed no SUI or pelvic organ prolapse. Laboratory analysis showed that her white blood cell count was 5.6 × 10^3^/mm^3^, hemoglobin was 12.5 g/dL, potassium was 3.4 mmol/L, alanine aminotransferase was 19 U/L, creatinine was 79 μmol/L, and serological tests were negative for hepatitis B surface antigen, anti-hepatitis C virus, and anti-HIV. Urine analysis showed microscopic hematuria, and urine culture was sterile. As part of the PFU examination, 2-dimensional (2D) ultrasound showed that the sling had a high position and was situated above the middle part of the urethra (Fig. 1A). The sling was locally folded in the left posterior urethra and partially under the bladder mucosa, where bladder stones had formed (Fig. 1B). Three-dimensional (3D) ultrasound showed the asymmetry of the sling, with the left side of sling slightly higher than the right side (Fig. 2). Ultrasonic 3D tomography showed that the left side of the sling was slightly higher than the right side, and part of the sling cut across the bladder mucosa at 5 o’clock above the left posterior part of the urethra (Fig. 2). The length of the submucosal sling was about 2 cm. Post-void residual volume was 100 mL.

The patient underwent lithotripsy and sling resection using a holmium laser under a cystoscope. Intraoperatively, the exposed sling and stone formation were found at the 5 o’clock position on the left side of the bladder wall (Fig. 1D). We used a holmium laser under a cystoscope for successful removal of bladder calculi and the erosion mesh along the bladder mucosa (Fig. 1E). The patient was discharged 4 days after surgery. The first week, 1-month, and 3-month follow-up visits showed a complete absence of recurrent urinary incontinence and other urinary complaints. The patient underwent follow-up pelvic ultrasound at 6 months, which showed no erosion mesh under the bladder mucosa (Fig. 1C).

Written informed consent was obtained from the patient for publication of this study, which was approved by the Ethics Committee of the author’s hospital.

## 3. Discussion and conclusions

Herein, we reported the case of a woman with SUI after the TOT procedure, who had persistent hematuria, frequency, and urgency. PFU was used to diagnose the erosion sling material and bladder stones in this case, which were successfully removed under a cystoscope. There was no recurrence or complications during the follow-up.

Regardless of the method used, the success rate of vaginal suspension surgery is over 90%. Besides, sling surgeries are routinely performed on patients with SUI, because the success rate of minimally invasive surgery is high, and the recovery rate is rapid. In 1996, Ulmsten et al first proposed the TVT method.^[4]^ However, in order to reduce significant complications after TVT, such as bladder and bowel damage, Delorme described the transobturator route in 2001.^[5]^ Although there are fewer complications for TOT procedures than for TVT procedures, complications such as bladder perforation have been reported after TOT surgery.^[6,7]^ The incidence of mesh placement in the bladder and urethra is estimated at 1 to 6%. There are several possible causes of erosion of sling mesh, including intraoperative perforation of bladder detrusor, improper incision, incision infection, rejection of sling, and poor nutritional status of the vaginal mucosa. The bladder, urethra, or vagina is subsequently eroded due to the material.^[8]^ In the present case, postoperative gross hematuria occurred early and lasted for 6 months. The possible reason for sling erosion in the patient might be due to partial intraoperative damage to the bladder wall. Bladder erosion usually results from chronic inflammation, and improper placement, folding or curling of the mesh can accelerate this complication.^[9]^ Although cystoscope was used for exploration during the operation, no signs of bladder injury were found because the injury did not penetrate the bladder wall. In addition, intraoperative cystoscopy did not find bladder perforation, which might lead to be misdiagnosed. That might be because the misplaced sling was close to the bladder neck, which could not be detected using the routine rigid cystoscope. In this case, ultrasound showed that the bilateral sling was asymmetrically placed, with the left side of sling higher than the right side. Moreover, bladder erosion of left sling was confirmed using a cystoscope. The left side of sling might have been overstrained by the surgeon. Thereafter, continuous compression of the mesh belt led to bladder ischemia, full-thickness necrosis, and subsequent erosion perforation.^[10]^ Therefore, it is necessary to carefully separate the tissue layers and monitor the tension of the sling during surgery. If injuries to the bladder or other parts are suspected during the procedure, intraoperative PFU can also be used to observe the location and shape of the sling in real-time and provide timely guidance for the surgical planning.

Currently, cystoscopy, magnetic resonance imaging (MRI), and ultrasound are the main methods to detect postoperative sling. The cystoscope can directly find the sling and hence it is the gold standard compared to other imaging examinations, but it is also invasive. Cystoscopy is suggested for cases with predisposing factors for complications or suspected perforation. However, it is not routinely possible to recognize the misplaced sling if it is very near to the bladder neck. Missed cases under cystoscopy have been reported.^[11]^ During the rigid cystoscopy, the use of 70° endoscope lenses is particularly essential to observe the bladder neck and avoid missed diagnosis. Although MRI is a noninvasive examination, the diagnosis rate of the polypropylene sling on MRI varies from 23.1 to 100%, which is closely related to the experience level of the diagnostic physician.^[12]^ So it is suitable for vaginal or cervical erosion, but not for adhesion and granulation thickening tissue. Pelvic floor 3D ultrasound examination of the lower urinary tract is a simple, rapid, dynamic, noninvasive, repeatable and radiation-free diagnostic method. Since the urethral sling shows high echo under ultrasound and the surrounding tissue shows low echo, ultrasound can more accurately and correctly display the artificial sling compared to other methods.^[13]^ We used 2D ultrasound to detect the bladder erosion on the left side of the sling, and 3D reconstruction showed asymmetry on both sides of the sling wherein the left side of mesh was slightly higher than the right side, because the left side of the sling had folded and passed over the bladder mucous membrane layer. In addition, according to 3D tomographic ultrasound imaging, ultrasonic diagnosis was consistent with the surgical results of this patient.

In summary, mesh erosion into the bladder is a rare complication of TOT surgery, but surgeons must consider the possibility of this complication. PFU can accurately show the position and shape of the sling, which is important for choosing surgical options.

## Acknowledgments

The authors would like to thank everyone who contributed to our study especially staff from the Urology Department, Hospital Jinhua.

## Author contributions

**Conceptualization:** Yelin Lou, Yibo Zhou.

Data curation: Yelin Lou.

Formal analysis: Yang Hu, Yibo Zhou.

Funding acquisition: Yelin Lou.

Investigation: Yibo Zhou.

Methodology: Yelin Lou, Yang Hu.

Software: Yang Hu.

Writing – original draft: Yelin Lou, Yang Hu, Yibo Zhou.

Writing – review & editing: Yelin Lou, Yang Hu, Yibo Zhou.
